# Case report: New is not always better: treatment of non-bacterial thrombotic endocarditis

**DOI:** 10.3389/fcvm.2023.1208190

**Published:** 2023-07-12

**Authors:** Elias Akiki, Ali Ahmad, Edward A. El-Am, Ana I. Casanegra, Kyle W. Klarich, Reto Kurmann

**Affiliations:** ^1^ Department of Cardiovascular Medicine, Mayo Clinic, Rochester, MN, United States; ^2^ Department of Internal Medicine, Saint Louis University School of Medicine, Saint Louis, MO, United States

**Keywords:** non-bacterial thrombotic endocarditis, anticoagulation, embolic stroke, heparin, warfarin, DOAC, apixaban

## Abstract

An elderly female with metastatic adenocarcinoma of the lung and atrial fibrillation presented with multiple embolic strokes while on anticoagulation with Apixaban. After further investigation, a TEE showed lesions of non-bacterial thrombotic endocarditis on the mitral valve. A decision to switch the patient to LMWH for anticoagulation was then made and a follow-up TEE showed resolution of the NBTE. In this abstract, we show that heparin should remain as the anticoagulation agent of choice in the setting of NBTE associated with malignancy.

## Introduction

Non-bacterial thrombotic endocarditis (NBTE) is a condition characterized by the formation of sterile vegetations on previously undamaged heart valves (1). Common risk factors for NBTE include autoimmune conditions and malignancies. One study on patients with underlying malignancy showed an NBTE prevalence of 19% (2). The hallmark feature of NBTE is recurrent systemic embolization most commonly presenting as a sudden neurologic deficit (1–3).

## Case report

### History of presentation

A 70-year-old female with metastatic lung adenocarcinoma presented to the Emergency Department with new-onset headaches and retrosternal chest pain radiating to the right arm.

### Past medical history

The patient had stage IV lung adenocarcinoma with metastases to the pleura, pericardium, and mediastinal lymph nodes. She was on maintenance therapy with pembrolizumab and pemetrexed. She also had a history of atrial fibrillation on apixaban and had undergone pericardiocentesis earlier that same year after presenting with cardiac tamponade with a malignant pericardial effusion.

### Investigations

A CT scan of the head done in the ED disclosed new brain hypodensities concerning for brain metastasis. A subsequent MRI showed changes consistent with new multifocal embolic infarcts throughout the cerebral and cerebellar hemispheres, largest in the left cerebellar hemisphere and right temporal lobe.

Due to the presence of new multiple embolic infarcts, a transthoracic echocardiography (TTE) was then obtained and showed the presence of a small 7–8 mm soft mobile echodensity attached to the atrial surface of the mitral leaflet tips (Supplementary Video S1). Because of the underlying malignancy, NBTE was suspected but a diagnosis of subacute bacterial endocarditis could not be ruled out in the setting of immunosuppression. Blood cultures came back negative, and a workup for antiphospholipid syndrome was negative as well. A transesophageal echocardiogram (TEE) was done at our institution redemonstrating the soft mobile echodensity at the tip of both leaflets in a “kissing-lesion” appearance and measuring up to 1.1 cm in width. Moderate mitral valve regurgitation was noted (Supplementary Video S2).

Laboratory tests revealed an elevated high-sensitivity troponin level of 364 (up from a baseline of 44 previously) alongside new lateral ST depression at V4–V6 and new T-wave inversions on ECG. To further investigate the presentation of atypical chest pain with elevated troponins, a cardiac MRI demonstrated signs of myopericarditis.

### Differential diagnosis

Brain Metastasis, NBTE, Subacute bacterial endocarditis, Myxoma, Papillary fibroelastoma, NSTEMI, Myocarditis.

### Management

The diagnosis of NBTE in the setting of a multifocal stroke with peri-infarct edema complicated the management plan of the patient due to the risk of hemorrhagic transformation of the ischemic stroke. The NBTE and subsequent strokes occurred despite adequate dosing and adherence to anticoagulation with apixaban. This was considered an apixaban failure.

**Figure 1:**
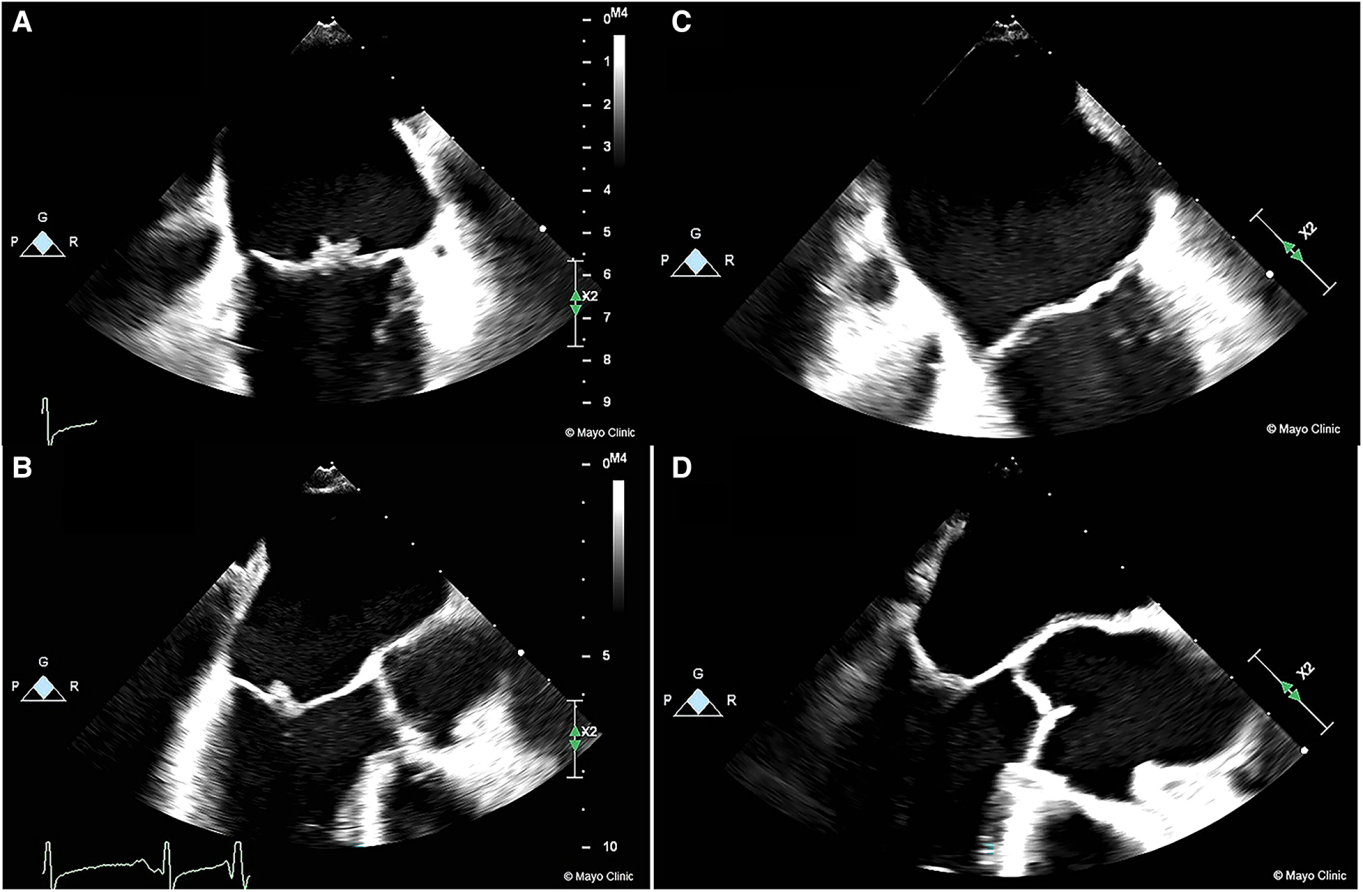
Images of NBTE seen on TEE at presentation prior to LMWH treatment (**1A/1B**) compared to TEE done 4 weeks after treatment showing complete resolution (**1C/1D**).

After careful review and consideration by the multidisciplinary team and shared-decision making with the patient, a decision was made to transition the patient to parenteral anticoagulation immediately due to the high risk of recurrent embolization in the setting of apixaban failure. The patient was started on a lower dose of enoxaparin initially (40 mg BID subcutaneous) due to the concern of hemorrhagic transformation. The dose was titrated up to 1 mg/Kg twice daily (70 mg). Anti-Xa levels were checked 4 h after the 4th dose and demonstrated therapeutic levels of 1.01. A repeat CT of the head was performed after 2 therapeutic doses (70 mg) of enoxaparin to assess for hemorrhagic conversion of infarcts. She was discharged on therapeutic low molecular weight heparin (LMWH) therapy, and a TEE repeated after 4 weeks of therapy showed complete resolution of the NBTE (Figure 1, Supplementary Video S3).

## Discussion

NBTE is an under-recognized complication in patients with known underlying malignancy. More specifically, patients with adenocarcinomas are at a higher risk of NBTE than patients with other types of malignancies (2.7% vs. 0.47% *p* < 0.05) (4). NBTE is usually asymptomatic until embolization occurs either to the brain or to other vascular beds.

This report presents the case of a patient with stage IV lung adenocarcinoma who presented with new embolic strokes despite therapeutic anticoagulation, which raises the suspicion of NBTE. Furthermore, our patient presented with atypical chest pain and elevated troponins. This should also raise the suspicion of NBTE microemboli to the coronary circulation. A CT cardiac angiogram ruled out large coronary emboli, but smaller emboli could be difficult to be visualized. Subsequently, a cardiac MRI demonstrated myopericarditis possibly caused by pemetrexed, which would explain the chest pain, elevated troponins and headaches (5). Patients with suspected NBTE should be evaluated for valvular vegetations with a TTE initially. If no definite diagnosis could be revealed with a TTE, the next step in suitable candidates would be to proceed with a TEE, which has been shown to be more sensitive for the detection of small vegetations (6). It is important to rule out infective endocarditis to confirm the diagnosis of NBTE. This process varies between institutions, but usually includes 2 to 3-sets of negative blood cultures. Once NBTE is suspected, antiphospholipid syndrome must be ruled out as an underlying cause. In our patient the negative blood cultures and antiphospholipid antibodies make the underlying malignancy the most likely etiology for NBTE.

Treatment guidelines for NBTE largely consist of treating the underlying condition and anticoagulation. In patients with advanced metastatic disease such as in our case, treating the underlying condition is not feasible. Like in a large subset of patients the mainstay of NBTE treatment revolves around anticoagulation (7). Current guidelines from the American College of Chest Physicians (ACP) recommend LMWH or unfractionated heparin for the treatment of NBTE and the prevention of thromboembolism (8). The patient discussed in this report was properly adhering to the appropriate Apixaban dose (5 mg bid) for prevention of embolization in the setting of atrial fibrillation. Apixaban was also proven to be efficient and safe in patients with atrial fibrillation and a history of cancer (9). No contemporary studies in the literature have evaluated the benefit of anticoagulation with warfarin in comparison to LMWH and unfractionated heparin. The ACP cites older studies that suggest that heparin might be superior to warfarin for the prevention of thromboembolism in NBTE patients. The first study from 1987 (10) did not directly compare heparin with warfarin and only studied 10 autopsy-proven cerebral embolization from NBTE who received anticoagulation. The second study from 1986 (11) extrapolated their therapy choice for NBTE from the treatment of Trousseau's syndrome as the authors believed that the two conditions formed a “continuum”. These studies exhibit methodological flaws and lack robust evidence to support the claim in question. The literature fails to address a very important topic when it comes to anticoagulating the NBTE patient and that is the efficacy of the direct oral anticoagulants (DOACs) vs. LMWH and unfractionated heparin. Recent studies have shown that DOACs are not inferior to LMWH in preventing recurrent venous thromboembolism (VTE) in cancer patients (12). Data regarding the efficacy of DOACs in NBTE is extremely limited but some recent case reports (13–15) have shown similar instances where DOACs have failed to prevent embolization in NBTE patients. This would be interesting to follow-up on in order to study potential NBTE development and ramifications in the setting of a cancer patient on DOAC.

## Conclusion

In summary, this case describes a case of NBTE presenting with embolic strokes despite anticoagulation with a direct factor Xa inhibitor. The NBTE vegetation resolved after switching to LMWH therapy. The treatment recommendations for NBTE in current guidelines rely on a restricted body of evidence and we hope this case could help improve our understanding of NBTE therapy in future studies.

## Data Availability

The original contributions presented in the study are included in the article/**Supplementary Material**, further inquiries can be directed to the corresponding author.

## Supplementary material

SUPPLEMENTARY VIDEO 1

Transthoracic echocardiography (TTE) showing initial mobile echodensity attached to the atrial surface of the mitral leaflet tips.

SUPPLEMENTARY VIDEO 2

Transesophageal echocardiography (TEE) showing the soft mobile echodensity at the tip of both leaflets in a “kissing-lesion” appearance.

SUPPLEMENTARY VIDEO 3

Transesophageal echocardiography (TEE) done 4 weeks after treatment with LMWH showing complete resolution of the NBTE vegetation.
